# Arsenic in Drinking Water and Human Biomonitoring in Eastern Croatia: An Exploratory Environmental Health Study

**DOI:** 10.3390/jox16050165

**Published:** 2026-09-01

**Authors:** Marina Vidosavljević, Domagoj Vidosavljević, Vlatka Gvozdić, Dinko Puntarić, Ivana Jelavić, Mario Šekerija, Mario Begović, Miroslav Venus, Lidija Kalinić

**Affiliations:** 1 Community Health Center, Osijek-Baranja County, Park Kralja Petra Krešimira IV 6, 31000 Osijek, Croatia; vidosavljevic.marina@gmail.com; 2 Interdisciplinary Postgraduate Study in Molecular Biosciences, Josip Juraj Strossmayer University of Osijek, 31000 Osijek, Croatia; 3 Faculty of Medicine Osijek, Josip Juraj Strossmayer University of Osijek, Cara Hadrijana 10 E, 31000 Osijek, Croatia; ijelavic@mefos.hr; 4 Department of Chemistry, Josip Juraj Strossmayer University of Osijek, Ulica Cara Hadrijana 8/A, 31000 Osijek, Croatia; vgvozdic@kemija.unios.hr; 5 Faculty of Medicine, Croatian Catholic University, Ilica 242, 10000 Zagreb, Croatia; 6 National Cancer Registry, Croatian Institute of Public Health, 10000 Zagreb, Croatia; mario.sekerija@hzjz.hr; 7 Andrija Štampar School of Public Health, School of Medicine, University of Zagreb, Rockefellerova 4, 10000 Zagreb, Croatia; 8 Faculty of Medicine, European University Brčko District, Bijeljinska bb, 76100 Brčko, Bosnia and Herzegovina; begovicvu@gmail.com; 9 Faculty of Dental Medicine and Health Osijek, Josip Juraj Strossmayer University of Osijek, Crkvena 21, 31000 Osijek, Croatia; venusmiroslav@gmail.com; 10 Department of Biology, Josip Juraj Strossmayer University of Osijek, Ulica cara Hadrijana 8/A, 31000 Osijek, Croatia

**Keywords:** arsenic, drinking water, human biomonitoring, urine, hair, serum, generalized linear model, environmental exposure, MDCK1, acetylcholinesterase, ecological analysis

## Abstract

Eastern Croatia includes settlements with historically elevated arsenic (As) concentrations in drinking water. Of 488 recruited adults, nine were excluded solely because no questionnaire was available; the exclusion was independent of measured arsenic concentrations, and the harmonised analytical cohort therefore comprised 479 adults from eight settlements. Total As concentrations in drinking water, hair, serum, and urine were measured in samples collected in June 2015 by inductively coupled plasma mass spectrometry. Kidney cancer data (ICD-10 C64) for 2000–2018 were obtained from the Croatian National Cancer Registry and analysed ecologically. MDCK1 cells were exposed to As_2_O_3_ at 10–200 µg/L for 48, 72, and 96 h, and acetylcholinesterase (AChE) activity was measured in human erythrocytes. Čepin had the highest median As concentrations in drinking water (191.75 µg/L) and urine (68.14 µg/L). Across all eight settlements, drinking-water As was strongly associated with urinary As (slope = 0.537; 95% CI, 0.352–0.722; R^2^ = 0.894; *p* < 0.001). However, in a sensitivity analysis excluding Čepin and using the seven non-Čepin settlement means reported, the association weakened (r = 0.740; R^2^ = 0.548; slope = 1.246; 95% CI, −0.054 to 2.547; *p* = 0.057), indicating substantial influence of the Čepin hotspot. Associations with hair As (R^2^ = 0.294; *p* = 0.165) and serum As (R^2^ = 0.239; *p* = 0.219) were not statistically significant. In a multivariable Gamma generalized linear model, residence in Čepin was associated with higher expected urinary As than residence in Vladislavci (adjusted mean ratio, 2.90; 95% CI, 1.90–4.42; *p* < 0.001), while female sex was associated with a lower expected concentration (adjusted mean ratio, 0.80; 95% CI, 0.66–0.97; *p* = 0.024). No statistically significant effect of As_2_O_3_ on MDCK1 viability or erythrocyte AChE activity was observed. The findings support urinary total As as a biomarker of recent environmental exposure, but do not establish causal associations with kidney toxicity or kidney cancer.

## 1. Introduction

Arsenic is a naturally occurring toxic metalloid and an important groundwater contaminant. Chronic exposure to inorganic arsenic through drinking water has been associated with adverse health outcomes, including cancers of the urinary tract. Parts of the Pannonian Basin, including eastern Croatia, are affected by geogenic arsenic release from alluvial sediments, resulting in elevated concentrations in groundwater and drinking-water supplies [1,2,3,4]. Concentrations reported in eastern Croatia have reached 611 µg/L, substantially exceeding the current European Union parametric value of 10 µg/L [4]. The kidney and urinary tract are relevant to arsenic toxicokinetics because arsenic metabolites are largely eliminated in urine. However, the magnitude and dose–response pattern of renal effects and kidney cancer risk remain uncertain, particularly at low-to-moderate environmental exposure levels [5,6,7].

Epidemiological studies of populations exposed to arsenic-contaminated drinking water have reported associations with chronic kidney disease, proteinuria, and end-stage kidney disease after adjustment for major confounders [8,9,10,11,12]. Experimental studies indicate that arsenic can induce oxidative stress, mitochondrial dysfunction, inflammation, and profibrotic signalling in renal tissue [13,14,15,16,17]. Nevertheless, differences in arsenic species, co-exposures, exposure duration, and outcome definitions complicate direct comparison across studies. The present study therefore aimed to characterise environmental arsenic exposure in eastern Croatia by combining drinking-water monitoring and human biomonitoring. Secondary objectives were to describe settlement-level patterns in cumulative crude C64 occurrence and to assess short-term effects of selected As_2_O_3_ concentrations on renal epithelial cell viability and erythrocyte AChE activity. The study did not measure clinical renal function, kidney injury biomarkers, or individual cancer outcomes.

## 2. Materials and Methods

### 2.1. Study Area

Sampling was conducted in five settlements in Osijek-Baranja County: Vladislavci, Čepin, Dalj, Osijek, and Našice. Našice is located in Osijek-Baranja County, but its water supply originates from the Papuk foothills and has not historically shown elevated arsenic concentrations. The remaining study settlements, including Osijek, are located within the Drava–Danube Basin, where elevated arsenic concentrations in groundwater have been reported [17].

**Figure 1:**
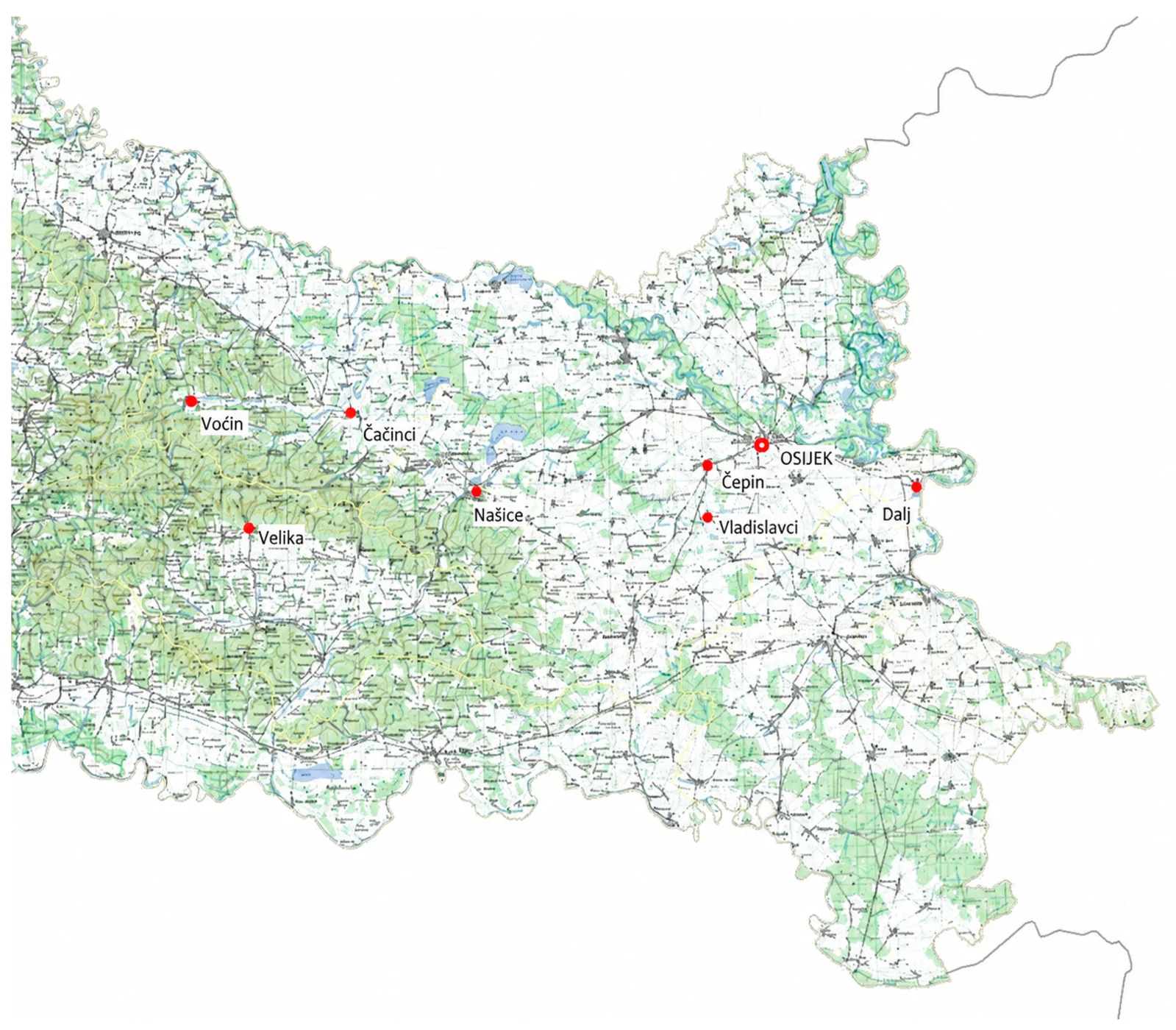
Map of the sampling locations.

Three reference settlements with low measured arsenic concentrations in drinking water were selected: Čačinci and Voćin in Virovitica-Podravina County and Velika in Požega-Slavonia County. The location of all sampling sites is shown in Figure 1.

### 2.2. Water Sampling and Analysis

Drinking-water samples were collected at 81 sampling sites during June 2015. Samples were collected in 250-mL polyethylene bottles that had been soaked in 10% HNO_3_ at room temperature for 24 h, packed in polyethylene bags, and filled at the sampling site. Samples were acidified on site with 10 mL/L of 65% nitric acid and stored at 4 °C until analysis. Total arsenic was measured; arsenic species were not determined.

### 2.3. Human Biomonitoring and Analytical Procedures

The original recruited cohort comprised 488 adults. Nine participants were excluded because they did not complete the study questionnaire and their biological measurements could not be linked reliably to the prespecified demographic and exposure covariates. The exclusion was based solely on missing questionnaire records and was independent of the measured arsenic concentrations; these participants were not classified or removed as statistical outliers. The final analytical cohort comprised 479 adults: 377 from Osijek-Baranja County (Osijek, n = 65; Našice, n = 81; Vladislavci, n = 88; Čepin, n = 40; and Dalj, n = 103), 80 from Virovitica-Podravina County (Čačinci, n = 49; and Voćin, n = 31), and 22 from Požega-Slavonia County (Velika, n = 22). All 479 participants completed the questionnaire covering sociodemographic characteristics, lifestyle factors, and possible occupational and wartime exposure to metals and metalloids. The same cohort is used in tables and in the individual-level multivariable analysis.

Data provenance and overlap with previous publications were assessed explicitly. Water and hair observations from 67 water-sampling sites and 377 participants in Vladislavci, Dalj, Čepin, Našice, and Osijek originated from the same 2015 project and were previously reported in the Water article [17], which also included Vinkovci; Vinkovci is not included in the present analysis. Water, urine, and 2000–2018 cancer-registry data for these five localities were also included in a previous ecological report [18]. The present study adds 14 water-sampling sites and 102 participants from the Papuk reference settlements Voćin, Čačinci, and Velika, serum measurements, a harmonised 479-participant analysis, the multivariable Gamma model, sensitivity regressions, the expanded PCA, and the MDCK1 and AChE experiments. Previously reported observations are therefore reanalysed within an expanded, integrated dataset and are not presented as newly generated data.

The study was conducted in accordance with applicable ethical principles. As part of an ongoing long-term project, the study was approved by the Ethics Committee of the Faculty of Medicine Osijek (No. 2158/61-02/82/2-06). All participants provided written informed consent.

For serum analysis, venous blood was collected by a laboratory technician, centrifuged, transferred to 3.8-mL cryotubes (TPP Techno Plastic Products AG, Trasadingen, Switzerland, and stored at −30 °C. After thawing, 10 mL of 1% HNO_3_ was added to 0.5 mL of serum. Samples were analysed by ICP-MS using an autosampler and 15-mL polypropylene tubes. Before use, the tubes were filled with 1% (*v*/*v*) HNO_3_ for at least 24 h and rinsed three times with 5 mL of the same solution.

Urine was collected from all participants in standard urine containers and analysed by ICP-MS (PerkinElmer, Waltham, MA, USA).

Hair was cut from the occipital region using stainless-steel scissors and stored in polyethylene bags for transport. In the laboratory, hair was washed with distilled water, soaked in acetone for 1 h, rinsed 10 times with deionised water, placed on filter paper, and air-dried for 24 h. Nitric acid was then added (0.1 g hair/1 mL of 65% HNO_3_), and the samples were left for 2 h before microwave digestion.

Total arsenic concentrations in water, urine, serum, and digested hair samples were quantified by inductively coupled plasma mass spectrometry using an ELAN DRC-e ICP-MS system (PerkinElmer, Waltham, MA, USA). The radiofrequency power was 1050 W. Argon (>99.99%) was used at plasma, auxiliary, and nebuliser flow rates of 15.00, 1.20, and 0.88 L/min, respectively. Polyatomic interferences affecting arsenic determination were reduced in the dynamic reaction cell using methane (CH_4_) as the reaction gas.

The instrument was calibrated after every 12 samples using the 71-Element Group Multi-Element Standard Solution (Inorganic Ventures, Christiansburg, VA, USA), with yttrium, indium, terbium, and bismuth used as internal standards. Calibration standards were prepared in 1% HNO_3_. Analytical performance was externally assessed through interlaboratory comparison with IFA Tulln and collaborating institutions in Vienna. Quality-control materials included ICP Multi Element Standard Solution X CertiPUR for Surface Water Testing (Merck, Darmstadt, Germany), Trace Elements Urine Blank and Trace Elements Urine (SERO AS, Billingstad, Norway), ClinChek Serum Control for Trace Elements (Levels I and II; RECIPE Chemicals + Instruments GmbH, Munich, Germany), Seronorm Trace Elements Serum (Levels I and II; Sero, Billingstad, Norway), human hair IAEA-086 (IAEA, Vienna, Austria), human hair NIES No. 13 (NIES, Tsukuba, Japan), and NIST SRM 1577a Bovine Liver. Analytical blanks were used to estimate detection limits as the mean blank response plus three standard deviations and quantification limits as the mean blank response plus ten standard deviations.

The matrix-specific LOD and LOQ values supplied in the analytical records were 0.06 and 0.18 µg/L for serum, 0.15 and 0.45 µg/L for urine, and 0.002 and 0.006 µg/g for hair, respectively. For water, the archived analytical records documented an LOD of 0.04 µg/L, but the corresponding water LOQ could not be recovered. The archived records also did not document a separate rule for measurements falling between the water LOD and LOQ; therefore, their treatment cannot be reconstructed retrospectively. Values below the documented LOD were replaced by LOD/√2. Very low water concentrations close to the detection limit should consequently be interpreted cautiously and not with greater quantitative certainty than the archived analytical documentation supports. Analysis of the CertiPUR reference solution yielded 52.2 µg/L against a certified value of 53.0 µg/L, corresponding to 98.49% recovery. Analyses were performed at the Andrija Štampar Teaching Institute of Public Health and the Institute for Medical Research and Occupational Health, Zagreb, Croatia. The archived documentation did not contain the complete calibration range and linearity coefficients, field-blank records, matrix-specific analytical precision or relative standard deviations, or measured-versus-certified values for all urine, serum, and hair reference materials; these parameters could not be reconstructed retrospectively and are acknowledged as a limitation.

### 2.4. Cancer-Registry Data and Exploratory Multivariate Analysis

Principal component analysis (PCA) was used solely as an exploratory multivariate visualisation of settlement-level patterns in arsenic concentrations in drinking water, urine, hair, and serum and cumulative crude kidney cancer occurrence. PCA was used to identify patterns of co-location and clustering; it was not used to test associations, estimate risk, or establish causality.

Data on newly registered malignant neoplasms of the kidney, except the renal pelvis (ICD-10 code C64), were obtained from the Croatian National Cancer Registry of the Croatian Institute of Public Health, Zagreb, for the period from 1 January 2000 to 31 December 2018. The registry extract available for this analysis contained sex-specific and total case counts aggregated over the complete 19-year period; year-specific counts by locality were not available. Registry cases were not linked to the biomonitoring participants.

Because the registry data covered a 19-year period, the population of each investigated administrative locality according to the 2011 Croatian Population Census was used as a fixed denominator [19]. For each locality, the cumulative number of registered cases was divided by the corresponding census population and expressed per 100,000 inhabitants. This descriptive approach was selected because of the small number of cases in several localities.

The cumulative crude C64 occurrence was calculated as follows:

Cumulative crude C64 occurrence per 100,000 = (registered C64 cases during 2000–2018/population  according to the 2011 Census) × 100,000.

These values represent cumulative crude descriptive measures over the complete 19-year period and should not be interpreted as annual incidence rates, age- or sex-standardised rates, cumulative incidence proportions in a closed cohort, or individual-level estimates of cancer risk.

### 2.5. Cell Culture

The Madin–Darby canine kidney cell line (MDCK1; ATCC CRL-2935; American Type Culture Collection, Manassas, VA, USA) was used to investigate the short-term effects of arsenic trioxide (As_2_O_3_) on renal epithelial cell viability. As_2_O_3_ was dissolved in sterile distilled water and tested at 10, 20, 50, 100, and 200 µg/L. Untreated control cells were cultured under identical conditions. Cells between passages 24 and 26 were maintained in Dulbecco’s modified Eagle medium (DMEM;Gibco, Thermo Fisher Scientific, Inchinnan, UK) supplemented with 10% heat-inactivated fetal bovine serum (FBS; Gibco, Thermo Fisher Scientific, Inchinnan, UK), 2 mM glutamine, and 100 U/mL penicillin–streptomycin. Cultures were maintained in a humidified atmosphere at 37 °C with 5% CO_2_ (IGO 150 CELLlife, JOUAN, Thermo Fisher Scientific, Waltham, MA, USA).

### 2.6. MTT Cell-Viability Assay

Cell-viability assays were performed using a slightly modified National Cancer Institute protocol [20,21]. MDCK1 cells were seeded in 96-well flat-bottom plates (Greiner, Frickenhausen, Austria) at 2 × 10^4^ cells/mL. Twenty-four hours later, cells were exposed to As_2_O_3_ (Sigma-Aldrich, St. Louis, MO, USA) at 10, 20, 50, 100, or 200 µg/L and incubated for 48, 72, or 96 h. Cell viability was measured by the MTT assay [20]. After removal of the growth medium, 50 µL of MTT solution (5 mg/mL) was added to each well. After 4 h of incubation at 37 °C, the water-insoluble formazan crystals were dissolved in 150 µL dimethyl sulfoxide (DMSO). Absorbance was measured at 595 nm using a microplate reader (iMark, Bio-Rad, Hercules, CA, USA). Each experiment was performed three times in triplicate.

Cell viability (%) = [(ODsample − ODbackground)/(ODcontrol − ODbackground)] × 100.

ODbackground was the optical density of MTT solution and DMSO without cells, whereas ODcontrol was the optical density of untreated cells cultured under the same conditions.

### 2.7. Acetylcholinesterase Activity Assay

The acetylcholinesterase assay was included as a complementary mechanistic experiment to evaluate whether arsenic trioxide could directly affect the activity of an enzyme involved in cholinergic neurotransmission and commonly used as a functional marker of neurotoxicity. The selected As_2_O_3_ concentrations were intended to establish a controlled ex vivo concentration–response relationship rather than to reproduce environmental or physiological exposure levels. Therefore, the assay should not be interpreted as a model of renal toxicity or as a direct simulation of population exposure. The absence of significant AChE inhibition under the tested conditions indicates that As_2_O_3_ did not directly inhibit the enzyme in this experimental system, but does not exclude other mechanisms of arsenic-induced neurotoxicity. Native intact human erythrocytes obtained from heparinised blood were used as the source of acetylcholinesterase (AChE). Erythrocytes were isolated by centrifugation (20 min, 2500 rpm), washed three times with saline to remove residual plasma, and diluted with 0.1 M sodium phosphate buffer (pH 7.4) to the original whole-blood volume. Samples were stored at −20 °C until analysis. Heparinised blood was obtained from healthy volunteers at the Institute for Medical Research and Occupational Health, Zagreb, with approval from the relevant Ethics Committee (Class 01-18/23-02-2/1; Reg. No. 100-21/23-16).

Acetylthiocholine iodide (ATCh; Sigma-Aldrich, St. Louis, MO, USA) was used as the AChE substrate in a 20 mM stock solution prepared in water. 5,5′-Dithiobis(2-nitrobenzoic acid) (DTNB; Sigma-Aldrich, USA) was prepared as a 6.0 mM solution in phosphate buffer for determination of enzyme activity by the Ellman method [22]. The reaction mixture contained 30 µmol/L DTNB, phosphate buffer, and erythrocytes at a final 400-fold dilution. Following addition of the erythrocyte enzyme source, As_2_O_3_ was added at final concentrations of 0.025, 0.0125, 0.00625, 0.00025, and 0.0002 mg/mL. No pre-incubation of AChE with As_2_O_3_ was performed before substrate addition. ATCh was added immediately to initiate the reaction, which was monitored for 2 min at 25 °C; thus, the effective observation period was 2 min. A control reaction containing As_2_O_3_ and substrate without enzyme was included to assess non-enzymatic substrate hydrolysis; no non-enzymatic reaction was observed. Absorbance was measured at 436 nm using a Cary UV-Vis Multicell Peltier spectrophotometer (Agilent, Santa Clara, CA, USA) [23].

### 2.8. Statistical Analysis

Continuous arsenic concentrations are presented as mean, median, minimum, maximum, standard deviation (SD), and 95% confidence interval (CI) for the mean. Settlement-level ordinary least-squares regressions used the arithmetic mean drinking-water As concentration as the independent variable and the corresponding arithmetic mean urine, hair, or serum As concentration as the dependent variable, with each settlement contributing one unweighted observation. Primary regressions included all eight settlements. Because Čepin was a markedly higher-exposure observation and could dominate the fitted relationships, sensitivity analyses were repeated after exclusion of Čepin. Regression equations, Pearson correlation coefficients where relevant, slopes with two-sided 95% CIs, R^2^ values, two-sided *p*-values for the slope, and 95% confidence bands are reported. For the urine sensitivity analysis, the seven non-Čepin observations are exactly the arithmetic settlement means reported in matching Tables. (water As, urine As; µg/L): Vladislavci (16.32, 35.69), Dalj (1.43, 27.48), Našice (0.13, 23.30), Osijek (23.60, 37.62), Voćin (2.35, 7.56), Čačinci (0.96, 0.80), and Velika (0.56, 0.35). The regression was fitted by ordinary least squares without weighting by the number of water samples or biomonitoring participants. Hair and serum sensitivity estimates were calculated analogously from the corresponding settlement means in matching Tables. Principal component analysis was applied to standardized settlement-level variables solely for exploratory visualization. MTT and AChE data were analyzed using one-way analysis of variance followed by Tukey’s honestly significant difference post hoc test. Experiments were repeated three times, with three to five technical replicates per condition, and results are presented as mean ± SD. Analyses were performed using Statistica 13.1 and 14.0.0.15 (TIBCO Software Inc., Palo Alto, CA, USA).

### 2.9. Multivariable Gamma Generalized Linear Model

An exploratory multivariable generalized linear model with Gamma distribution and log link was fitted to total urinary arsenic concentration (µg/L), which was positive and right-skewed. Sex, age group (19–39, 40–59, and ≥60 years), and residential settlement were entered simultaneously as prespecified fixed effects. Male sex, age 19–39 years, and Vladislavci were the reference categories. Exponentiated coefficients are reported as adjusted mean ratios (aMRs) with two-sided 95% CIs calculated using HC3 heteroscedasticity-consistent standard errors. The global settlement effect was evaluated using a Wald test.

The analysis used the same final cohort of 479 participants included in the biomonitoring tables. The nine recruited participants without questionnaires were excluded before modelling because the prespecified covariates could not be assigned; no participant was excluded on the basis of arsenic concentration. Household-specific drinking-water arsenic, individual water consumption, urinary creatinine or specific gravity, arsenic speciation, dietary arsenic, and detailed occupational exposure measurements were unavailable. The model therefore estimates adjusted geographic and demographic differences in total urinary arsenic and should not be interpreted as an individual causal dose–response model.

## 3. Results

### 3.1. Arsenic Concentrations in Drinking Water and Human Biomonitoring

Table 1 summarises arsenic concentrations in drinking water across the eight study settlements. Čepin had the highest mean, median, and maximum concentrations (184.34, 191.75, and 250.40 µg/L, respectively). Osijek also had consistently elevated concentrations (mean 23.60 µg/L; median 24.17 µg/L). In Vladislavci, the mean concentration (16.32 µg/L) exceeded 10 µg/L because of a single high value (160.16 µg/L), whereas the median was low (1.33 µg/L), indicating marked within-settlement heterogeneity. All summary measures in Dalj, Našice, Voćin, Čačinci, and Velika were below 10 µg/L.

The biomonitoring results in Table 2, Table 3 and Table 4 show distinct exposure profiles across the settlements. Hair arsenic concentrations were highest in Vladislavci and Čepin, with elevated values also observed in Dalj, whereas substantially lower concentrations were found in Našice, Osijek, Voćin, Čačinci, and Velika. Because hair may be affected by external deposition from water, dust, and cosmetic products, these findings should be interpreted as supportive rather than definitive evidence of chronic internal exposure.

Urinary arsenic concentrations were highest in Čepin (median 68.14 µg/L; mean 111.98 µg/L), followed by Osijek, Dalj, and Vladislavci. The elevated urinary arsenic concentrations observed in Dalj and Našice despite lower water concentrations suggest that individual exposure may also reflect diet, mobility, occupational or agricultural activities, and other environmental sources. Serum arsenic concentrations were higher in the five Osijek-Baranja County settlements than in Voćin, Čačinci, and Velika, but the differences were less pronounced than those observed in urine.

Overall, the results indicate that urine provided the clearest settlement-level differentiation in relation to arsenic concentrations in drinking water. Hair supported evidence of longer-term exposure in several rural settlements, but the potential for external contamination limits interpretation. Serum showed the weakest geographical discrimination, consistent with its short biological half-life and greater intra-individual variability.

**Figure 2:**
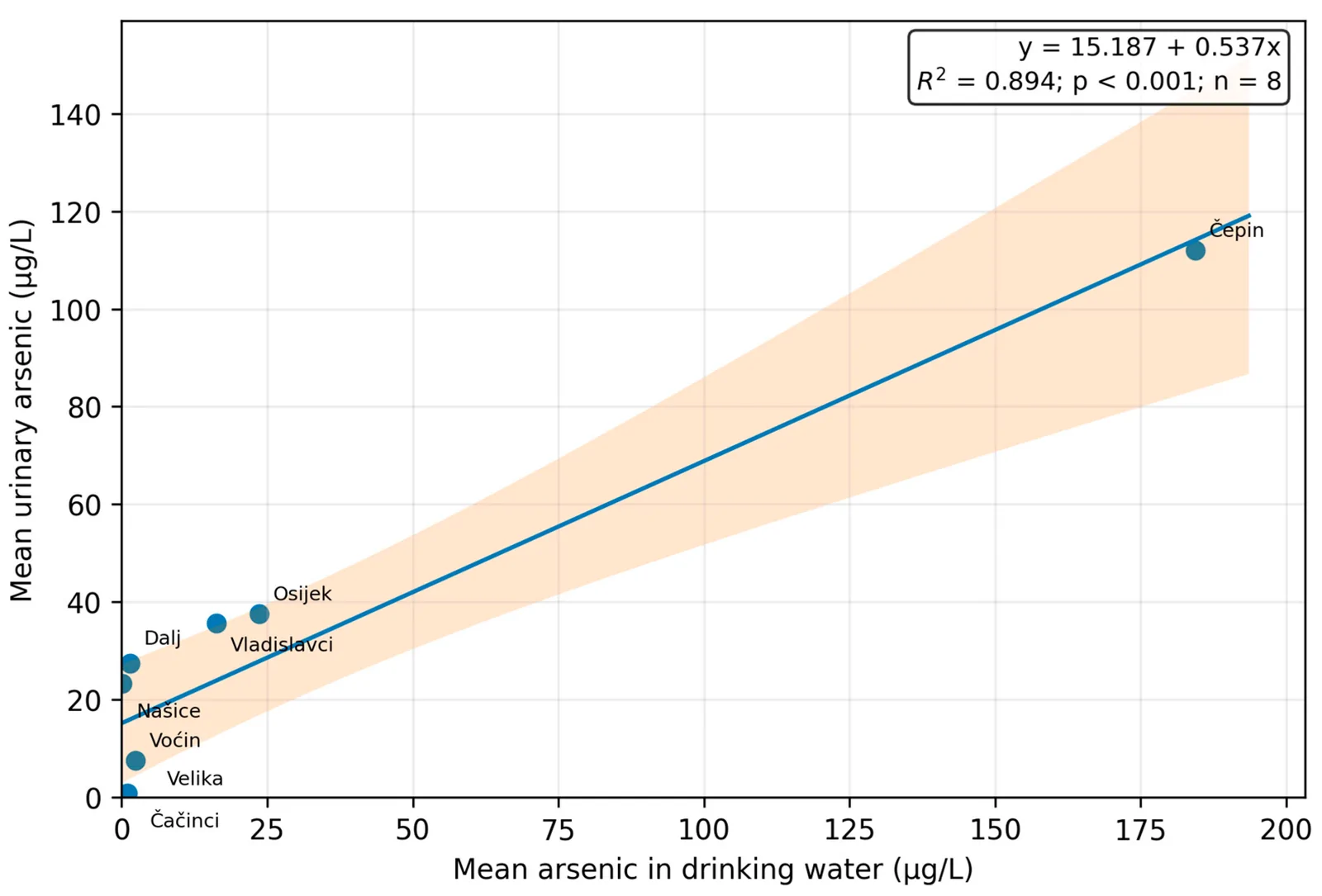
Settlement-level linear regression of mean urinary arsenic on mean drinking-water arsenic. The solid line represents the fitted ordinary least-squares regression and the shaded area the 95% confidence band (n = 8 settlements).

Across all eight settlements, mean drinking-water As was strongly associated with mean urinary As (y = 15.187 + 0.537x; slope = 0.537; 95% CI, 0.352–0.722; R^2^ = 0.894; *p* < 0.001) (Figure 2).

**Figure 3:**
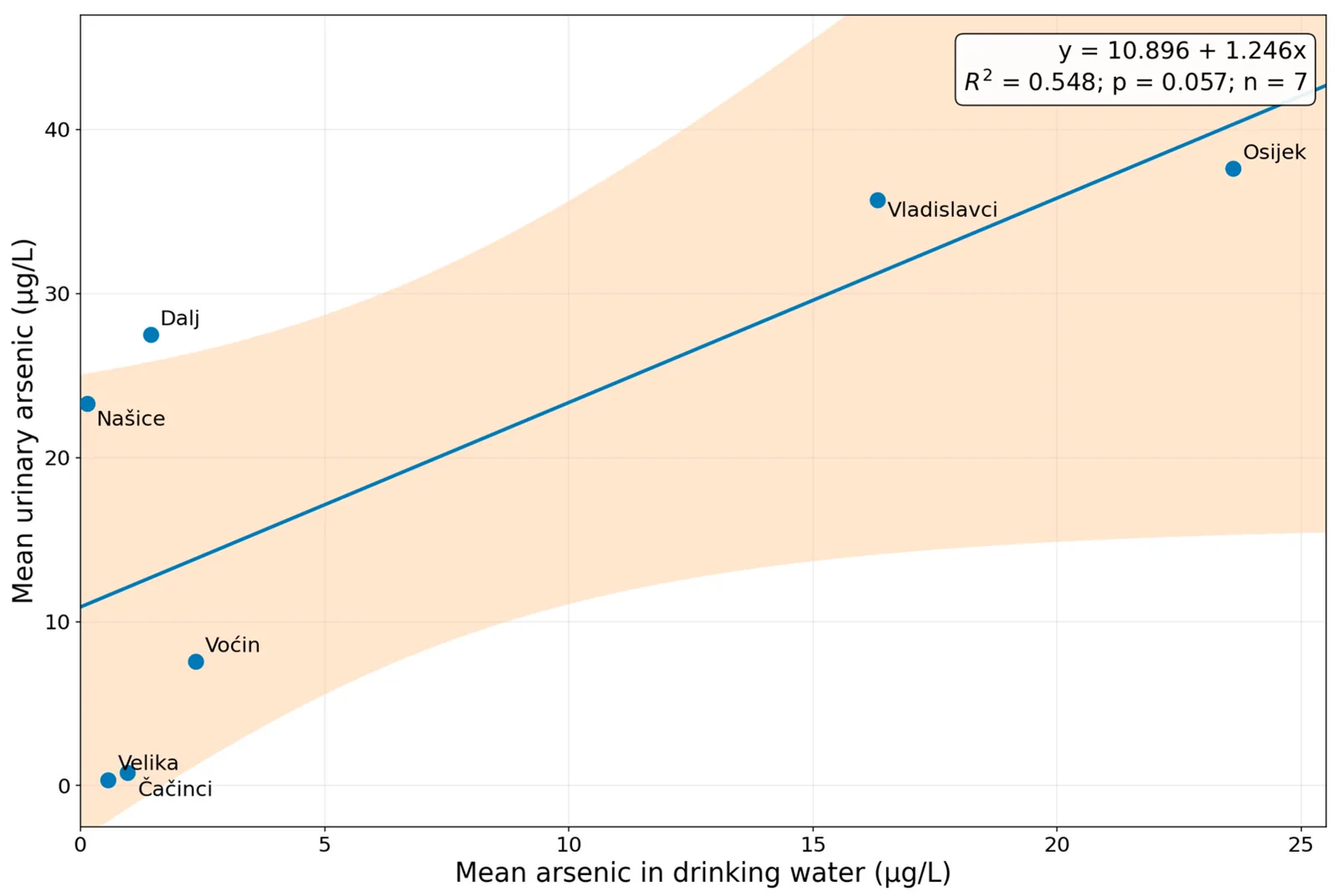
Sensitivity analysis of the settlement-level linear regression of mean urinary arsenic on mean drinking-water arsenic after exclusion of Čepin. The seven plotted observations are the arithmetic settlement means reported in Table 1 and Table 3. The solid line represents the fitted ordinary least-squares regression and the shaded area the 95% confidence band (n = 7 settlements).

In the sensitivity analysis excluding Čepin shown in Figure 3, recalculation from the seven non-Čepin settlement means reported in Table 1 and Table 3 produced a positive but weaker association (y = 10.896 + 1.246x; r = 0.740; R^2^ = 0.548; slope = 1.246; 95% CI, −0.054 to 2.547; *p* = 0.057; n = 7). The association was not statistically significant, although the remaining data indicated a moderate positive relationship between As concentrations in drinking water and urine. This result indicates that the strong full-sample association was substantially influenced by the Čepin hotspot. The sensitivity analysis is exploratory and does not establish nephrotoxicity, causality, or an individual-level exposure–response relationship.

**Figure 4:**
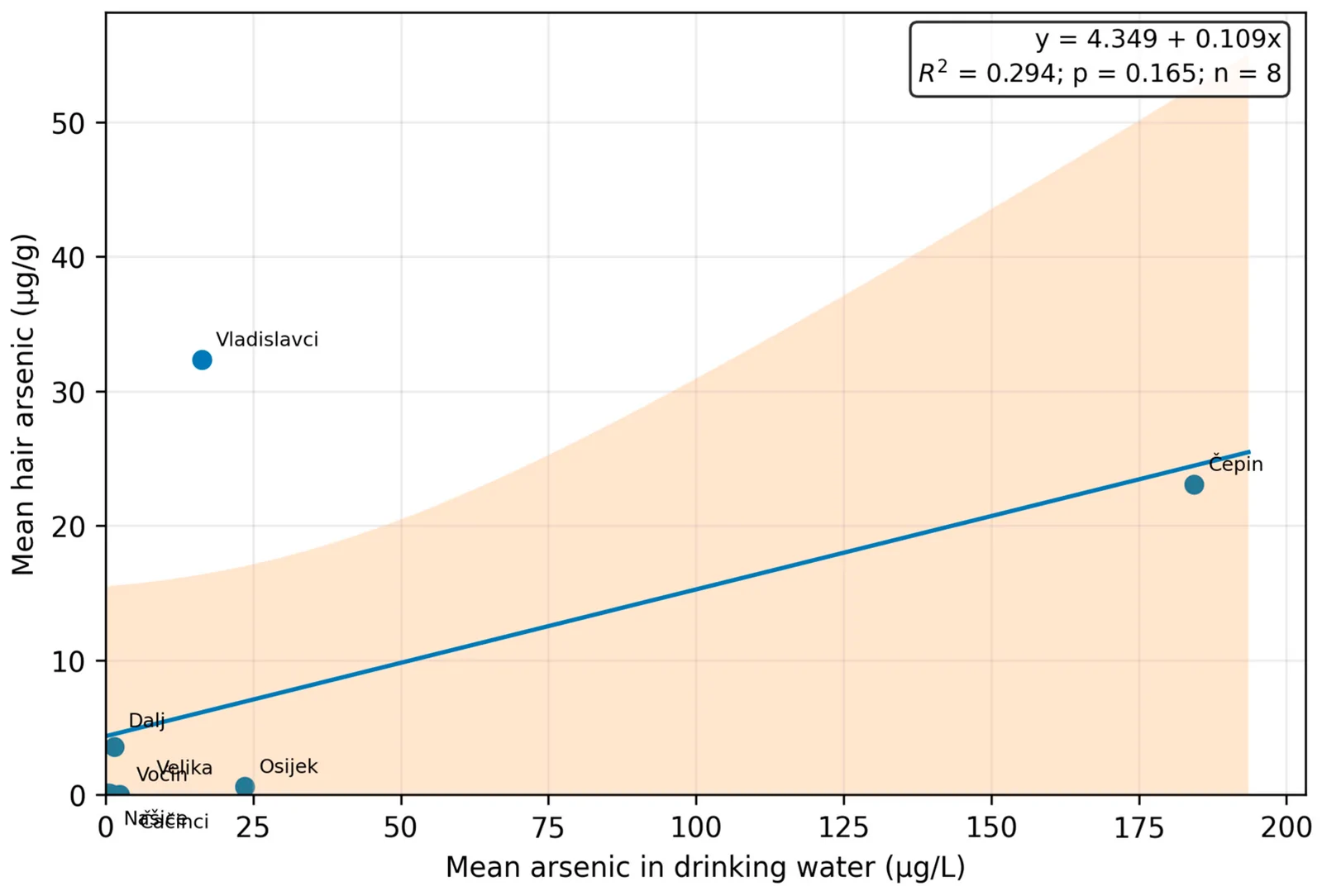
Settlement-level linear regression of mean hair arsenic on mean drinking-water arsenic. The solid line represents the fitted ordinary least-squares regression and the shaded area the 95% confidence band (n = 8 settlements).

Figure 4 shows a positive but statistically non-significant association between settlement mean drinking-water As and mean hair As across all eight settlements (y = 4.349 + 0.109x; β = 0.109; 95% CI, −0.060 to 0.278; R^2^ = 0.294; *p* = 0.165). After exclusion of Čepin, the association remained non-significant (β = 0.575; 95% CI, −0.725 to 1.874; R^2^ = 0.205; *p* = 0.307; n = 7). Hair concentrations may reflect longer-term exposure but are also susceptible to external contamination and non-water sources.

**Figure 5:**
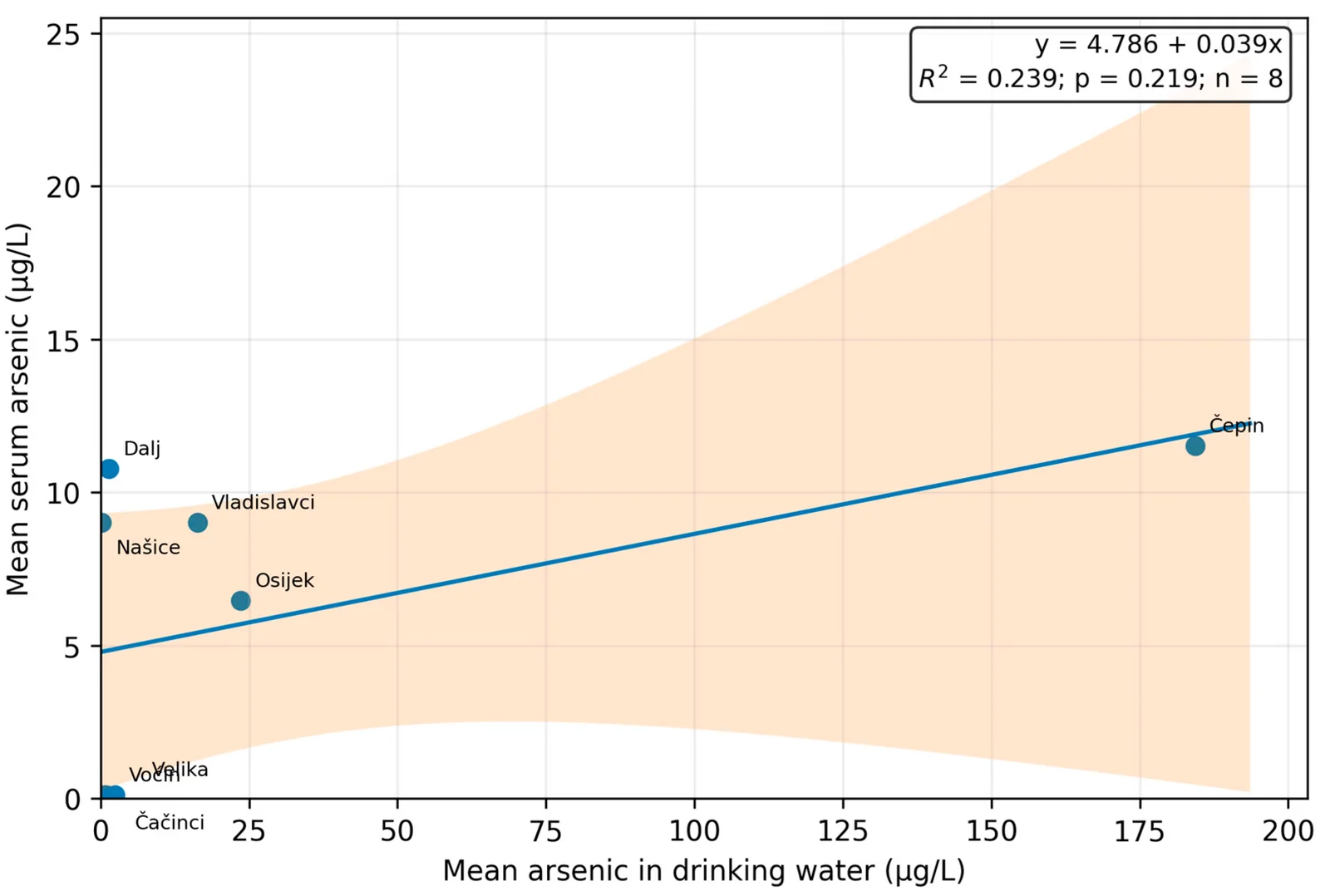
Settlement-level linear regression of mean serum arsenic on mean drinking-water arsenic. The solid line represents the fitted ordinary least-squares regression and the shaded area the 95% confidence band (n = 8 settlements).

Figure 5 shows a positive but statistically non-significant association between settlement mean drinking-water As and mean serum As across all eight settlements (y = 4.786 + 0.039x; β = 0.039; 95% CI, −0.030 to 0.107; R^2^ = 0.239; *p* = 0.219). After exclusion of Čepin, the association remained weak and non-significant (β = 0.160; 95% CI, −0.396 to 0.715; R^2^ = 0.099; *p* = 0.493; n = 7). Serum As was therefore less informative than urinary As for differentiating recent settlement-level exposure in this dataset.

### 3.2. Multivariable Gamma Generalized Linear Model of Urinary Arsenic Concentrations

The individual-level analysis presented in Table 5. included the final analytical cohort of 479 participants; the nine recruited participants without questionnaires were not included because the prespecified covariates could not be assigned. This exclusion was based solely on missing questionnaire records and was independent of measured arsenic concentrations. In the multivariable Gamma model, urinary arsenic concentrations differed significantly by residential settlement after adjustment for sex and age group (overall Wald χ^2^ = 823.30, df = 7; *p* < 0.001).

Compared with participants residing in Vladislavci, expected urinary arsenic was higher in Čepin (aMR = 2.90; 95% CI, 1.90–4.42; *p* < 0.001) and lower in Našice (aMR = 0.68; 95% CI, 0.48–0.94; *p* = 0.022), Voćin (aMR = 0.20; 95% CI, 0.11–0.35; *p* < 0.001), Čačinci (aMR = 0.021; 95% CI, 0.012–0.039; *p* < 0.001), and Velika (aMR = 0.009; 95% CI, 0.006–0.015; *p* < 0.001). Differences for Dalj and Osijek were not statistically significant.

Female participants had a lower expected urinary arsenic concentration than males (aMR = 0.80; 95% CI, 0.66–0.97; *p* = 0.024). Neither the 40–59-year nor the ≥60-year age group differed significantly from the 19–39-year reference group.

Residential settlement was strongly associated with urinary arsenic after demographic adjustment. Because individual household-water measurements, urinary dilution adjustment, arsenic speciation, dietary intake, and detailed exposure histories were unavailable, the model should be interpreted as an exploratory biomonitoring analysis rather than evidence of causation.

### 3.3. Descriptive Kidney Cancer Occurrence and Exploratory Principal Component Analysis

The absolute number of registered C64 cases, the corresponding 2011 census population, and the cumulative crude C64 occurrence for 2000–2018 are presented in Table 6. Osijek had the largest absolute case count (n = 261), reflecting its substantially larger population. Because several localities had small populations and few registered cases, the cumulative crude values are statistically unstable and should be interpreted as descriptive contextual measures rather than direct comparisons of cancer risk. For Dalj, registry data were available at the level of the Municipality of Erdut, which includes Dalj.

**Figure 6:**
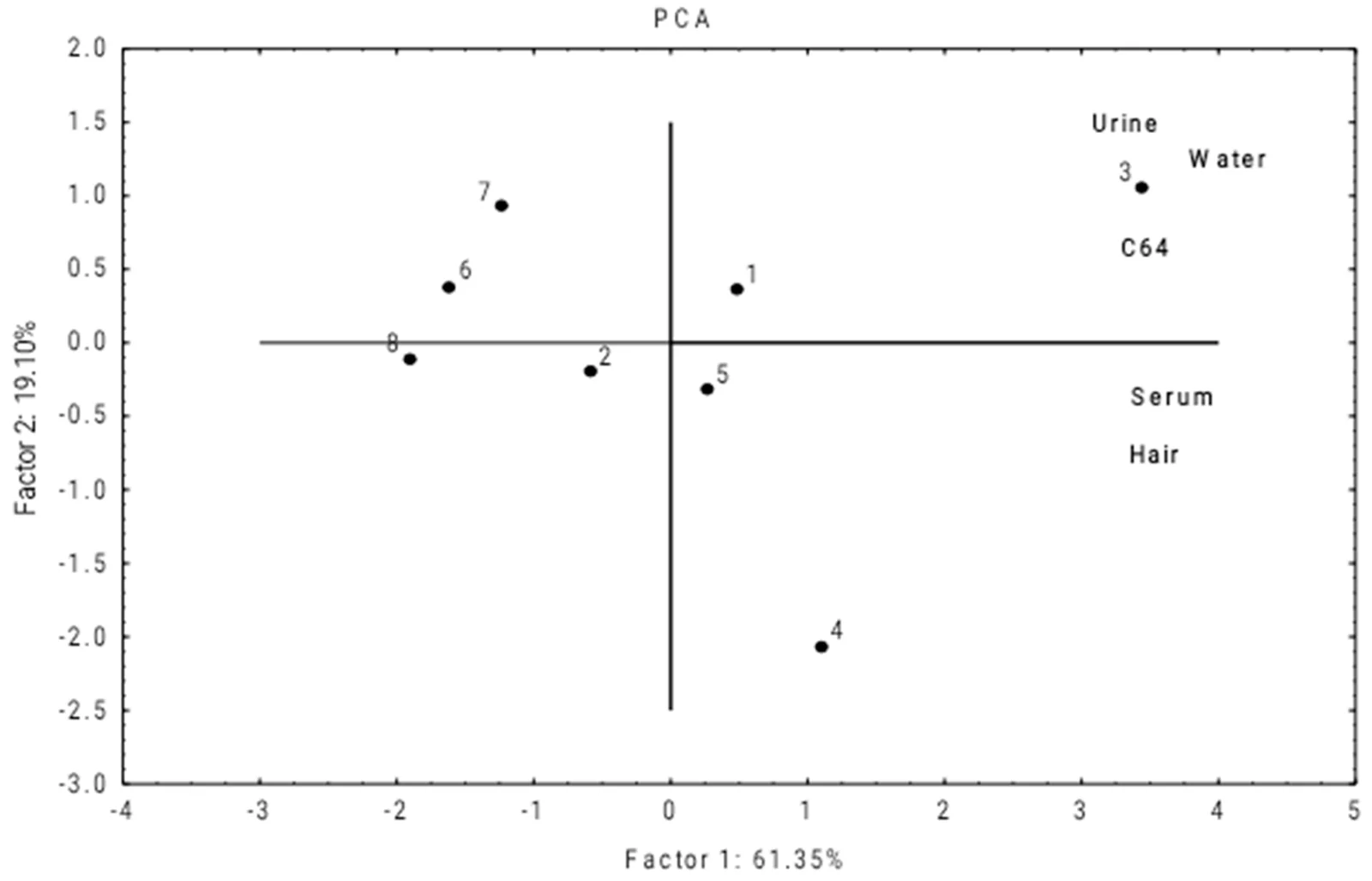
Exploratory principal component analysis of settlement-level arsenic concentrations in water and biological matrices and cumulative crude C64 occurrence.

The PCA results are presented as a biplot in Figure 6. The first two principal components explained approximately 80% of the total variance, allowing the data to be visualised in two dimensions. Three visual groupings are apparent: Voćin, Velika, and Čačinci cluster in the upper-left quadrant; Osijek, Našice, Vladislavci, and Dalj occupy the central region; and Čepin forms a separate single-settlement group. Čepin is positioned in the direction of the water and urinary arsenic vectors and the cumulative crude C64 occurrence vector. This proximity is a descriptive pattern of co-location and is not a statistical test of association or evidence of causality.

The biplot does not demonstrate a uniform relationship between arsenic concentrations in water or biological matrices and cumulative crude C64 occurrence across all settlements. This is particularly apparent in the low-arsenic settlements at the foothills of Papuk. Accordingly, the PCA is hypothesis-generating only and cannot resolve individual exposure, latency, smoking, occupational history, comorbidity, age, sex, or other potential confounders.

### 3.4. Effects of As_2_O_3_ on MDCK1 Cell Viability and AChE Activity

**Figure 7:**
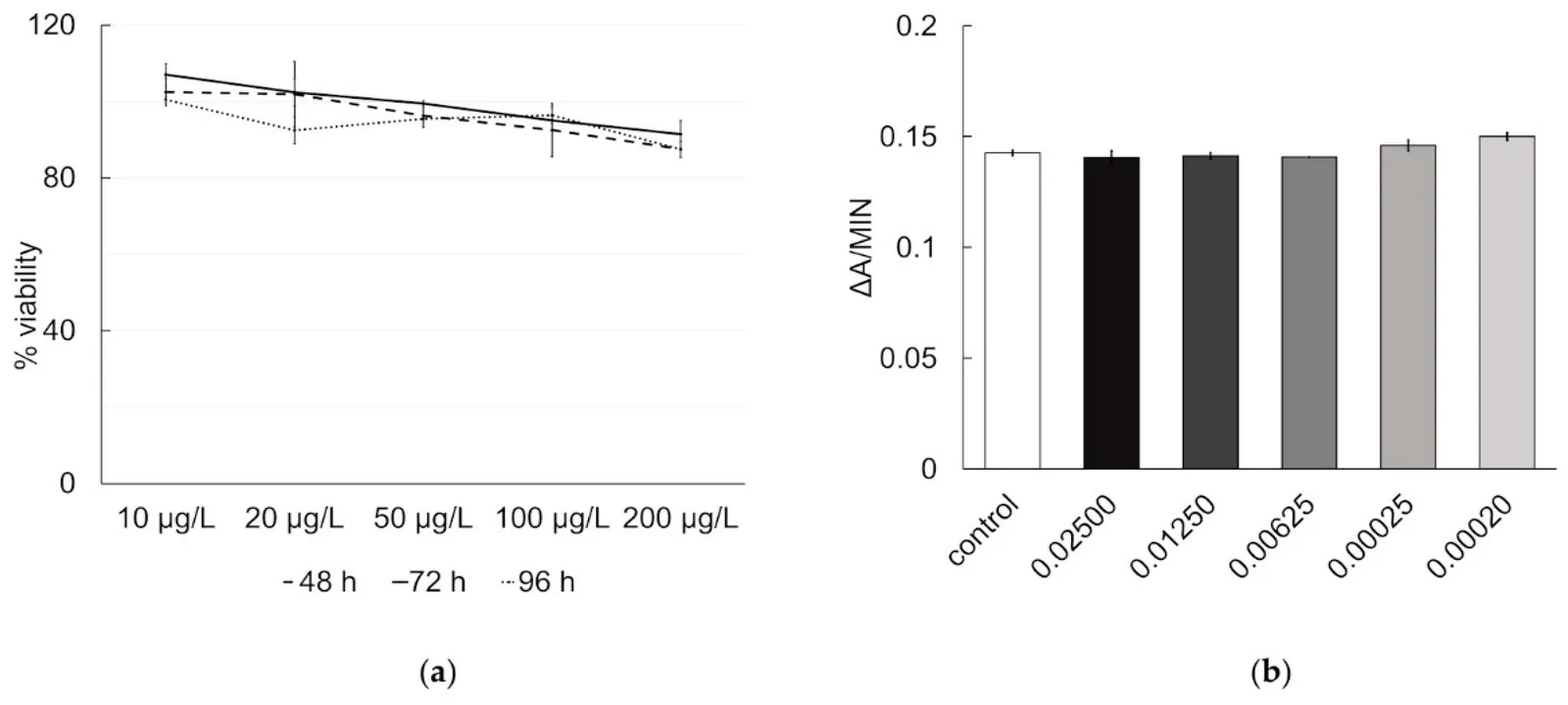
(**a**) MTT assay in MDCK1 cells after 48, 72, and 96 h of exposure to 10, 20, 50, 100, and 200 µg/L As_2_O_3_ and (**b**) acetylcholinesterase activity in intact human erythrocytes after exposure to 0.025, 0.0125, 0.00625, 0.00025, and 0.0002 mg/mL As_2_O_3_. Results are presented as mean ± SD. No significant between-group differences were detected by Tukey’s HSD test.

As_2_O_3_ did not produce a statistically significant concentration- or time-dependent effect on MDCK1 cell viability at 48, 72, or 96 h (Figure 7a). Likewise, no statistically significant change in erythrocyte AChE activity was observed at the tested concentrations (Figure 7b). These short-term experiments assess cell viability and erythrocyte enzyme activity only; they do not evaluate chronic nephrotoxicity, renal fibrosis, or carcinogenic transformation.

## 4. Discussion

Water analysis identified three settlements in which the mean arsenic concentration exceeded the European Union parametric value of 10 µg/L: Čepin, Osijek, and Vladislavci. Čepin was the most clearly high-exposure settlement, with a mean water arsenic concentration of 184.34 µg/L and a median of 191.75 µg/L. The high mean in Vladislavci was driven by a single elevated sample, whereas its median concentration was low. Čepin therefore represents the most consistent environmental hotspot in the present study, with elevated arsenic concentrations in drinking water and biological matrices. The local drinking-water supply was subject to a legal restriction because of high arsenic concentrations in 2009 [17].

The regional context is important for interpretation. During Croatia’s transition to the current European Union drinking-water standard, a temporary derogation allowed higher arsenic concentrations in certain supplies until 2019. Earlier monitoring in eastern Slavonia reported concentrations up to 611.89 µg/L in drinking water and elevated arsenic concentrations in hair [6]. These observations are consistent with the known hydrogeochemistry of the Drava–Danube Basin and underline the need for continued surveillance of water supplies. In a previous study in eastern Croatia, a reduction in arsenic concentrations after a change in water source was accompanied by lower arsenic concentrations in hair [17].

The present hair data are broadly consistent with previous reports of elevated hair arsenic in populations exposed to arsenic-contaminated drinking water [24,25,26,27]. Vladislavci and Čepin had the highest hair concentrations in the current study, and Dalj also showed elevated values. Hair may provide useful supportive evidence of longer-term exposure, but external contamination from water, dust, and cosmetic products can materially affect measurements. The higher concentrations observed in rural settlements may therefore reflect combined water, dietary, agricultural, and other environmental sources rather than drinking water alone. Hair analysis should therefore be interpreted cautiously [28].

Urinary arsenic showed the clearest relationship with drinking-water arsenic, whereas serum was less informative. This pattern is biologically plausible because urine reflects recent arsenic elimination, while blood arsenic is more transient and may be influenced by dietary organic arsenic species [27,28,29,30]. The highest urinary arsenic concentrations were observed in Čepin, consistent with its markedly elevated drinking-water arsenic concentration. However, the elevated urinary concentrations in Dalj and Našice also demonstrate that biomonitoring results should be interpreted in the context of potential non-water exposures.

The settlement-level regressions reinforce these exposure-assessment observations. Across all eight settlements, the water–urine association was strong and statistically significant (slope = 0.537; 95% CI, 0.352–0.722; R^2^ = 0.894; *p* < 0.001). After exclusion of Čepin and recalculation from the same settlement means reported in Table 1 and Table 3, the association weakened and was not statistically significant (r = 0.740; R^2^ = 0.548; slope = 1.246; 95% CI, −0.054 to 2.547; *p* = 0.057). The contrast demonstrates that the full-sample relationship was substantially influenced by the Čepin hotspot and should not be interpreted as a uniform exposure–biomarker gradient across eastern Croatia. The hair and serum regressions were non-significant both in the full eight-settlement analyses and after exclusion of Čepin. All settlement-level models are based on only seven or eight aggregated observations and are descriptive and hypothesis-generating rather than confirmatory. The matrices represent different exposure windows and are differently affected by external contamination, metabolism, and short-term variation.

The individual-level Gamma model included sex, age group, and residential settlement. After adjustment, residence in Čepin was associated with a 2.90-fold higher expected urinary As concentration than residence in Vladislavci. Female sex was associated with a 20% lower expected concentration, whereas neither age group was independently associated with urinary As. The geographic contrasts are directionally consistent with the marked differences in drinking-water As, but cannot establish a causal individual dose–response relationship because household water As, individual intake, urinary dilution, dietary exposure, and arsenic species were unavailable.

The biomonitoring findings identify arsenic exposure and excretion patterns; they do not establish renal injury, chronic kidney disease, or causality. No creatinine, eGFR, albuminuria, proteinuria, KIM-1, NGAL, or other clinical renal endpoint was measured in the present study. The kidney and urinary tract remain clinically relevant target systems because inorganic arsenic and its metabolites are eliminated in urine, and chronic exposure has been associated with urinary tract malignancy and kidney disease in epidemiological studies. Arsenic and inorganic arsenic compounds are classified as carcinogenic to humans, with established evidence for several cancer sites, including the urinary bladder [31].

To examine environmentally relevant concentrations in vitro, MDCK1 cells were exposed to trivalent inorganic arsenic (As_2_O_3_) at 10, 20, 50, 100, and 200 µg/L. No reduction in cell viability was observed after 48, 72, or 96 h of exposure. This negative result should be interpreted in the context of the experimental design: the assay assessed short-term effects on viability in a single cell line and does not model chronic exposure, arsenic biotransformation, oxidative injury, fibrosis, or carcinogenesis. Thus, absence of an acute cytotoxic effect at these concentrations does not exclude renal toxicity or carcinogenic potential after long-term exposure.

Epidemiological estimates of kidney cancer risk and short-term in vitro viability assays address different questions and should not be interpreted as directly comparable. Recent meta-analytic evidence has reported increasing estimates of kidney cancer risk with higher arsenic concentrations in drinking water [32]. Such findings may reflect prolonged exposure, latency, co-exposures, and outcome classification, whereas the MDCK1 experiment assessed only acute cellular viability. The contribution of upper urinary tract urothelial tumours and other diagnostic classifications should also be considered when interpreting registry-based kidney cancer data [33].

No significant change in erythrocyte AChE activity was observed. The assay assessed the immediate effect of As_2_O_3_ without pre-incubation, over a 2-min observation period after substrate addition. Previous studies have reported both increases and decreases in AChE activity after arsenic exposure in animal and aquatic models [34,35,36,37,38]. Differences in species, arsenic species and concentration, co-exposures, pre-incubation conditions, and experimental endpoints may explain these inconsistent findings. The present result therefore indicates only that the selected conditions did not measurably alter erythrocyte AChE activity during the short observation period; it does not exclude delayed, irreversible, haematological, oxidative, or other neurotoxic effects of arsenic.

Although the tested arsenic concentrations did not affect MDCK1 viability or erythrocyte AChE activity, the exploratory PCA placed Čepin in the direction of higher water and urinary arsenic concentrations and higher cumulative crude C64 occurrence. This descriptive pattern does not support an inference that arsenic contributed to kidney cancer risk in this study. Ecological analyses cannot link individual exposure to individual disease and are vulnerable to confounding, differential population structure, changes in exposure over time, and latency-related bias.

International experience demonstrates the public-health importance of geogenic arsenic contamination in groundwater. Major exposure episodes have been reported in Taiwan, Chile, Bangladesh, India, and the Pannonian Basin, including southern Hungary [39,40,41,42,43,44,45,46,47]. The geological similarity between eastern Croatia and neighbouring areas of the Danube Basin provides a plausible environmental context for the elevated concentrations observed in some local water supplies.

Quantitative risk assessment at low-to-moderate arsenic concentrations remains contested. Regulatory limits have been based on extrapolation from high-exposure populations, whereas alternative analyses have proposed different dose–response models and threshold assumptions [47]. The current European Union drinking-water standard of 10 µg/L reflects a precautionary public-health approach rather than a threshold below which all health effects can be excluded.

Meta-analyses and mechanistic studies have produced heterogeneous estimates of risk at lower exposure levels. Some analyses have suggested evidence of increased risk at concentrations below 50 µg/L, whereas others have not detected a clear association for specific cancer outcomes below that range [48,49,50,51]. These differences likely reflect variation in arsenic speciation, co-exposures, latency, study design, confounder control, and outcome ascertainment. Accordingly, the available evidence is better interpreted as a continuum of risk than as proof of a single universal threshold.

At substantially higher concentrations, chronic arsenic exposure is associated with clear adverse dermatological, cardiovascular, neurological, haematological, and cancer-related consequences [6,51,52]. The very high concentrations reported historically in some eastern Croatian supplies therefore remain relevant even where current remediation has reduced exposure.

The long latency of arsenic-associated cancer is also relevant. Follow-up studies in Chile have reported persistently elevated lung, bladder, and kidney cancer mortality decades after arsenic concentrations in drinking water were reduced [53,54,55,56]. This temporal pattern supports the need to consider historical exposure when interpreting contemporary cancer-registry data.

Systematic reviews of arsenic in drinking water and urinary tract cancer have generally found stronger evidence at higher exposure levels but have also highlighted uncertainty at lower concentrations [54]. The magnitude of any risk depends on cumulative exposure, arsenic species, duration, latency, and host susceptibility.

The inconsistent threshold estimates reported across studies underscore the importance of local exposure assessment and biomonitoring rather than reliance on a single concentration cut-off. This approach is particularly important in regions such as eastern Croatia, where arsenic concentrations vary substantially between nearby settlements and even between sampling sites within the same settlement.

Our previous ecological study of arsenic in drinking water, urinary arsenic, and urinary tract malignancies in eastern Croatia similarly generated a hypothesis of a possible association at concentrations lower than those reported in some high-exposure cohorts [18]. Together, these ecological observations justify further research but cannot substitute for an individual-level design with arsenic speciation, repeated biomonitoring, detailed confounder assessment, and longitudinal follow-up.

### Study Limitations

This study has several limitations. First, the cancer analysis was ecological and locality-based; registry cases were not linked to biomonitoring participants. The C64 values were cumulative crude measures using a fixed 2011 census denominator and were not age- or sex-standardised, and year-specific locality counts were unavailable. Second, the settlement-level regressions and PCA included only seven or eight localities. The strong full-sample water–urine regression was substantially influenced by Čepin, and all ecological models are exploratory. Third, nine of 488 recruited participants were excluded solely because no questionnaire was available and the prespecified covariates could not be assigned. Their exclusion was independent of arsenic concentration; they were not removed as statistical outliers. Fourth, the Gamma model lacked household-specific water measurements, individual intake, urinary dilution adjustment, arsenic speciation, dietary exposure, and detailed occupational histories. Fifth, clinical renal outcomes and renal injury biomarkers were not measured. Sixth, total arsenic was measured without speciation, and hair may be affected by exogenous contamination. Matrix-specific LOD and LOQ values were available for the biological matrices, and the water LOD was documented, but the water LOQ and any separate handling rule for measurements between the water LOD and LOQ could not be recovered from the archived records. In addition, the archived records did not contain complete calibration ranges, linearity coefficients, field-blank documentation, matrix-specific precision, or measured-versus-certified results for every biological matrix. Consequently, very low water concentrations close to the detection limit should be interpreted cautiously. Finally, the in vitro experiments assessed short-term MDCK1 viability and immediate erythrocyte AChE activity without pre-incubation and cannot model chronic exposure, metabolism, fibrosis, delayed enzyme effects, or carcinogenesis. These limitations require cautious interpretation and preclude causal inference.

## 5. Conclusions

Arsenic exposure remains a relevant environmental health concern in eastern Croatia. Čepin had the highest drinking-water and urinary arsenic concentrations. The full eight-settlement water–urine regression was strong, but after exclusion of Čepin the association weakened and was not statistically significant (R^2^ = 0.548; slope = 1.246; 95% CI, −0.054 to 2.547; *p* = 0.057), demonstrating substantial influence of the Čepin hotspot. In the multivariable Gamma model of the harmonised 479-participant cohort, residential settlement remained strongly associated with urinary As after adjustment for sex and age group; residence in Čepin was associated with higher expected urinary As than residence in Vladislavci, while female sex was associated with a lower expected concentration. Hair provided supportive but non-specific evidence of longer-term exposure, whereas serum was less informative. No short-term effect of As_2_O_3_ on MDCK1 viability or immediate erythrocyte AChE activity was observed at the tested concentrations. The PCA identified descriptive co-location of higher exposure markers and cumulative crude C64 occurrence in Čepin, but the ecological cancer data, limited number of localities, absence of standardization and individual clinical endpoints, and incomplete individual exposure data preclude causal inference about kidney cancer or kidney toxicity. Repeated biomonitoring and longitudinal studies with household-level exposure assessment, urinary dilution adjustment, arsenic speciation, renal biomarkers, and detailed confounder measurement are required.

## Figures and Tables

**Table 1:** Arsenic concentrations in drinking water by study locality (µg/L).

SD	Maximum	Minimum	Median	Mean	N	Location
40.06	160.16	0.33	1.33	16.32	16	Vladislavci
2.21	8.85	0.14	0.52	1.43	19	Dalj
53.82	250.40	30.60	191.75	184.34	12	Čepin
0.18	0.59	0.04	0.07	0.13	8	Našice
7.83	37.61	12.30	24.17	23.60	12	Osijek
1.91	4.60	0.59	2.08	2.35	4	Voćin
0.29	1.09	0.30	0.33	0.96	6	Čačinci
0.25	0.90	0.30	0.52	0.56	4	Velika

SD, standard deviation. Water data for Vladislavci, Dalj, Čepin, Našice, and Osijek were reported previously [17,18]; the Papuk reference settlements Voćin, Čačinci, and Velika are newly integrated in the present expanded analysis. The archived records documented a water LOD of 0.04 µg/L but did not contain a recoverable water LOQ; values at or near the detection limit should therefore be interpreted cautiously.

**Table 2:** Arsenic concentrations in hair by study locality (µg/g).

95% CI for Mean		SD	Max	Min	Median	Mean	N	Location
Upper	Lower							
52.67	12.01	95.94	647.35	0.060	0.37	32.34	88	Vladislavci
4.97	2.22	7.02	38.21	0.004	0.88	3.59	103	Dalj
51.47	1.37	88.87	421.76	0.018	1.04	23.05	40	Čepin
0.11	0.06	0.12	0.70	0.004	0.05	0.09	81	Našice
0.91	0.32	1.18	8.50	0.030	0.31	0.62	65	Osijek
0.04	0.02	0.02	0.11	0.006	0.02	0.03	31	Voćin
0.05	0.03	0.04	0.28	0.007	0.04	0.04	49	Čačinci
0.15	0.02	0.14	0.71	0.014	0.05	0.09	22	Velika

CI, confidence interval; SD, standard deviation. Hair observations for Vladislavci, Dalj, Čepin, Našice, and Osijek were reported previously [17]; observations from Voćin, Čačinci, and Velika are newly integrated here.

**Table 3:** Arsenic concentrations in urine by study locality (µg/L).

95% CI for Mean		SD	Max	Min	Median	Mean	N	Location
Upper	Lower							
45.62	25.76	46.86	294.50	0.28	24.16	35.69	88	Vladislavci
31.16	23.81	18.80	85.57	0.09	27.68	27.48	103	Dalj
148.93	75.03	115.52	351.51	0.87	68.14	111.98	40	Čepin
27.96	18.63	21.08	150.80	0.92	19.29	23.30	81	Našice
43.81	31.43	24.97	135.27	1.55	32.38	37.62	65	Osijek
11.80	3.32	11.55	52.84	0.18	3.34	7.56	31	Voćin
1.31	0.30	1.74	12.35	0.10	0.43	0.80	49	Čačinci
0.48	0.23	0.27	0.96	0.11	0.23	0.35	22	Velika

CI, confidence interval; SD, standard deviation. Urinary As observations for Vladislavci, Dalj, Čepin, Našice, and Osijek were included in a previous ecological report [18]; observations from Voćin, Čačinci, and Velika and the harmonised analyses are new to the present study.

**Table 4:** Arsenic concentrations in serum by study locality (µg/L).

95% CI for Mean		SD	Max	Min	Median	Mean	N	Location
Upper	Lower							
10.88	7.165	8.78	44.26	0.93	6.06	9.02	88	Vladislavci
13.20	7.94	12.30	52.86	0.06	4.75	10.78	103	Dalj
15.40	7.65	12.11	48.02	0.83	6.72	11.53	40	Čepin
10.35	7.67	6.05	28.02	1.17	6.30	9.01	81	Našice
8.23	4.69	7.14	48.15	0.76	5.02	6.46	65	Osijek
0.14	0.10	0.06	0.44	0.10	0.10	0.12	31	Voćin
0.11	0.10	0.01	0.19	0.10	0.10	0.11	49	Čačinci
0.13	0.10	0.04	0.25	0.10	0.10	0.12	22	Velika

CI, confidence interval; SD, standard deviation. Serum As measurements across the eight settlements were not reported in the previous Water or Acta Clinica Croatica publications [17,18].

**Table 5:** Multivariable Gamma generalized linear model of factors associated with urinary arsenic concentrations (n = 479).

*p*	95% CI	aMR	Category	Variable
—	—	Reference	Male	**Sex**
0.024	0.66–0.97	0.80	Female	
—	—	Reference	19–39	**Age group (years)**
0.822	0.81–1.30	1.03	40–59	
0.674	0.84–1.32	1.05	≥60	
—	—	Reference	Vladislavci	**Residential settlement**
0.108	0.58–1.06	0.78	Dalj	
<0.001	1.90–4.42	2.90	Čepin	
0.022	0.48–0.94	0.68	Našice	
0.976	0.71–1.39	0.99	Osijek	
<0.001	0.11–0.35	0.20	Voćin	
<0.001	0.012–0.039	0.021	Čačinci	
<0.001	0.006–0.015	0.009	Velika	

aMR, adjusted mean ratio; CI, confidence interval. Gamma generalized linear model with log link and HC3 robust standard errors. Sex, age group, and settlement were entered simultaneously. Reference categories were male sex, age 19–39 years, and Vladislavci. The overall settlement effect was significant (Wald χ^2^ = 823.30, df = 7; *p* < 0.001).

**Table 6:** Number of registered kidney cancer cases and cumulative crude C64 occurrence in the study localities, 2000–2018.

Cumulative Crude Occurrence Per 100,000 Inhabitants	Registered C64 Cases, n	Population According to the 2011 Census, n	Study Locality or Administrative Unit
241.56	261	108,048	Osijek
160.26	26	16,224	Našice
172.43	20	11,599	Čepin
123.15	9	7308	Municipality of Erdut, including Dalj
159.40	3	1882	Vladislavci
83.96	2	2382	Voćin
0.00	0	2802	Čačinci
231.85	13	5607	Velika

Data represent the total number of registered malignant neoplasms of the kidney, except the renal pelvis (ICD-10 code C64), during the period from 1 January 2000 to 31 December 2018. Population denominators were obtained from the 2011 Croatian Population Census. The cumulative crude occurrence was calculated as the total number of registered cases divided by the corresponding 2011 census population and multiplied by 100,000. These values are descriptive measures for the complete 19-year period and should not be interpreted as annual, age-standardised, or sex-standardised incidence rates. For Dalj, cancer-registry data were available at the level of the Municipality of Erdut, which includes Dalj. Values for the five Osijek-Baranja localities were included in a previous ecological report [18]; Voćin, Čačinci, and Velika are newly integrated in the expanded analysis. All locality labels, population denominators, case counts, and calculated values were rechecked against the source table; Čačinci had 0 cases and Velika had 13 cases.

## Data Availability

The original contributions presented in this study are included in the article. Further inquiries can be directed to the corresponding author.
